# Patricia Goldman-Rakic: A Pioneer in Cognitive Neuroscience

**DOI:** 10.7759/cureus.67915

**Published:** 2024-08-27

**Authors:** Deepti Avasthi

**Affiliations:** 1 Internal Medicine, St. Vincent Mercy Medical Center, Toledo, USA

**Keywords:** medical researc\her, brain mapping, cognitive science, pioneer in medicine, historical vignette

## Abstract

Patricia Goldman-Rakic (1937-2003) was a trailblazing neuroscientist whose groundbreaking work greatly advanced our understanding of the prefrontal cortex and its crucial role in higher cognitive functions like working memory and executive function. Her innovative research, which integrated anatomical, electrophysiological, and behavioral methods, provided foundational insights into the neural basis of cognition. This review underscores her significant contributions, personal challenges, and the enduring influence of her work in the field of neuroscience.

## Introduction and background

Patricia Goldman-Rakic was a pioneering neuroscientist whose groundbreaking research profoundly advanced our understanding of the brain’s prefrontal cortex, particularly its role in working memory. Born on April 22, 1937, in New York City, she devoted her career to exploring the brain’s intricate structure and function, challenging prevailing paradigms, and revealing the neural foundations of higher cognitive processes. Throughout her distinguished career, Goldman-Rakic received numerous accolades and was widely recognized as a leader in her field, shaping the direction of neuroscience for generations. Her untimely passing in 2003 left a lasting impact on the scientific community, with her contributions continuing to inspire ongoing research into memory and cognition.

Early life and education

Patricia Shoer was born in Salem, Massachusetts, United States, in 1937. From a young age, her curiosity about the natural world was evident, as she often spent hours engrossed in science books, dreaming of making her own discoveries. Her path to becoming Patricia Goldman-Rakic began at Vassar College, where she majored in psychology and graduated with honors in 1959.

Her quest for knowledge led her to the University of California, Los Angeles, where she pursued a PhD in developmental psychology. In 1963, she earned her doctorate, laying the foundation for a career that would span decades and profoundly influence generations of scientists. Her postdoctoral work at the National Institute of Mental Health (NIMH) further shaped her research trajectory [1].

During her postdoctoral work at NIMH, Patricia met and married Paul Rakic, a fellow neuroscientist. Balancing her burgeoning career with her personal life was challenging, but their shared passion for science created a strong partnership. Patricia’s life was marked by extraordinary professional achievements and the everyday challenges of being a woman in science during a predominantly male-dominated era.

Patricia’s career flourished as she explored the complexities of the prefrontal cortex. Her meticulous mapping of this brain region and its role in cognitive functions was groundbreaking. She identified distinct areas within the prefrontal cortex and demonstrated their specific roles in processes such as working memory and executive function.

## Review

Key contributions

Mapping the Prefrontal Cortex

Goldman-Rakic’s meticulous mapping of the prefrontal cortex was pioneering. She identified distinct regions within the prefrontal cortex and demonstrated their specific roles in cognitive processes [2]. Her research with nonhuman primates revealed the dorsolateral prefrontal cortex (DLPFC) as a crucial area for working memory, a finding that was later corroborated by studies in humans [3].

Working Memory

One of Goldman-Rakic’s most significant contributions was elucidating the neural mechanisms of working memory. She proposed that working memory depends on persistent neural activity in the prefrontal cortex [4]. Using single-cell recording techniques, Goldman-Rakic demonstrated that neurons in the DLPFC exhibited sustained activity during delay periods in working memory tasks [5]. This persistent activity was interpreted as the neural basis for maintaining information over short periods, a fundamental aspect of cognitive function.

Dopamine and the Prefrontal Cortex

Goldman-Rakic also explored the role of neurotransmitters in the prefrontal cortex, with a particular focus on dopamine. She discovered that dopaminergic projections to the prefrontal cortex modulate cognitive functions and that disruptions in this system could underlie various psychiatric disorders [6]. Her work provided a neural basis for understanding cognitive deficits observed in conditions such as schizophrenia and attention deficit hyperactivity disorder [7].

Cortical Microcircuits

Goldman-Rakic’s studies on the microcircuitry of the prefrontal cortex were groundbreaking. By combining neuroanatomical tracing, electrophysiology, and behavioral experiments, she described the intricate connections within the prefrontal cortex and between it and other brain regions [8]. This work laid the foundation for understanding how complex cognitive functions emerge from the interaction of neural circuits.

Influence on Psychiatric Disorders

Goldman-Rakic’s research had profound implications for psychiatry. By linking specific cognitive deficits to dysfunctions in prefrontal cortical circuits, she provided a framework for understanding the neural basis of psychiatric disorders [9]. Her insights have informed the development of therapeutic strategies aimed at ameliorating the cognitive impairments associated with these conditions [10].

Methodological innovations

Goldman-Rakic was renowned for her methodological rigor and innovation. She was among the first to combine neuroanatomical, electrophysiological, and behavioral methods to study the prefrontal cortex. This integrative approach enabled her to make comprehensive and impactful discoveries that would have been difficult to achieve using a single technique.

Honors and awards

Throughout her illustrious career, Goldman-Rakic received numerous prestigious honors for her groundbreaking contributions to neuroscience. In 1989, she was elected to the National Academy of Sciences for her pioneering work on the prefrontal cortex and working memory [11]. She served as President of the Society for Neuroscience from 1989 to 1990, highlighting her leadership and influence in the field [12]. In 1991, she was awarded the Lieber Prize for Schizophrenia Research by the Brain & Behavior Research Foundation for her work on the neural basis of schizophrenia [13]. Her significant contributions to cognitive science were further recognized with the Fyssen International Prize in Cognitive Science in 2002 [14]. Posthumously, she received the Distinguished Scientific Contribution Award from the American Psychological Association in 2003 for her outstanding contributions to psychology and neuroscience.

Testimonials from prominent figures

Goldman-Rakic’s influence extended far beyond her own research. Many prominent figures in neuroscience and related fields have praised her contributions and legacy:

*Patricia was a giant in the field of neuroscience. Her work on the prefrontal cortex and working memory has fundamentally changed our understanding of the brain. She was a mentor and inspiration to countless scientists* - Dr. Daniel Weinberger, Director of the Lieber Institute for Brain Development

*Patricia’s integrative approach to studying the brain was revolutionary. She combined different methodologies to provide a comprehensive understanding of the prefrontal cortex. Her work continues to influence research in cognitive neuroscience and psychiatry* - Dr. Amy Arnsten, Professor of Neuroscience at Yale University

Legacy and impact

Goldman-Rakic’s work has left an indelible mark on neuroscience. Her discoveries about the prefrontal cortex and working memory are cornerstones of our understanding of cognitive neuroscience. She trained and mentored numerous students and postdoctoral fellows who have continued to advance in the field. Her influence extends beyond her own research, as she played a pivotal role in shaping the direction of cognitive neuroscience as a discipline.

## Conclusions

Goldman-Rakic’s pioneering research on the prefrontal cortex and working memory has profoundly and permanently impacted neuroscience. Her integrative approach, combining anatomical, electrophysiological, and behavioral techniques, set a new standard for research in the field. Her insights into the neural basis of cognitive functions and their disruption in psychiatric disorders continue to guide current research and therapeutic strategies. Goldman-Rakic’s legacy is marked by innovation, rigor, and a deep commitment to unraveling the complexities of the brain.
